# Recurrent coronary artery in-stent restenosis caused by metal allergy: a case report

**DOI:** 10.3389/fcvm.2025.1693234

**Published:** 2026-01-09

**Authors:** Yang Li, Zhengbang Dong, Qiming Dai, Yi Feng, Lijuan Chen, Genshan Ma

**Affiliations:** 1 Department of Cardiology, Zhongda Hospital, School of Medicine, Southeast University, Nanjing, China; 2 Department of Dermatology, Zhongda Hospital, School of Medicine, Southeast University, Nanjing, China

**Keywords:** allergy, drug-coated balloon, metal, recurrent in-stent restenosis, skin patch test

## Abstract

This article describes the case of a middle-aged woman who developed recurrent in-stent restenosis (ISR) due to metal allergy. The first ISR occurred after implantation of a nickel-rich Medtronic Endeavor Resolute stent (35% nickel, 35% cobalt, and 20% chromium). A skin patch test revealed a strong nickel allergy (++), with no allergic reaction to cobalt. Nickel allergy was considered to be the cause of the first ISR. Then, a drug-coated balloon platinum–chromium alloy stent was implanted, and ISR occurred again during follow-up. The article discusses this special case and clinically relevant management options. This case highlights a scenario with refractory in-stent restenosis persisting despite various treatment strategies, including stents with different metal components, drug-coated balloons, and intensive drug therapy.

## Introduction

In-stent restenosis (ISR) is the main limitation of percutaneous coronary stenting, irrespective of a history of metal allergy (1–6). Metal allergy, a delayed hypersensitivity reaction, has been proposed as one of the mechanisms of recurrent ISR. Nickel allergy is a common cause of metal allergy leading to recurrent ISR (7). This report describes a case of a middle-aged woman who developed recurrent ISR, possibly due to metal allergy.

## Case presentation

**Figure 1:**
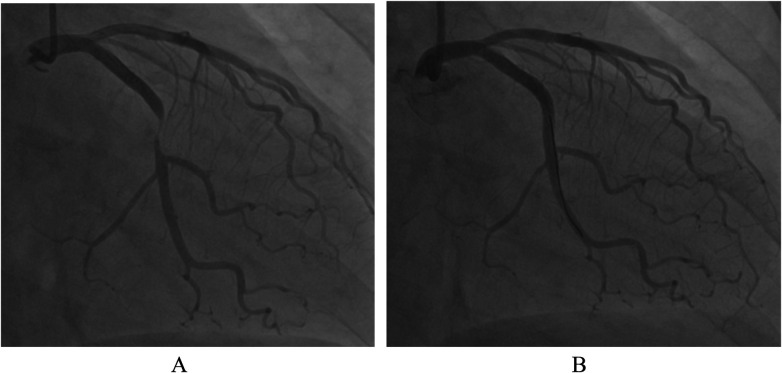
**(A)** CAG before PCI. **(B)** CAG after stenting.

A 48-year–old woman was repeatedly admitted to the hospital due to acute coronary syndrome (ACS). In November 2019, she was admitted to our department for the first time due to unstable angina. Coronary angiography (CAG) revealed mild stenosis in the proximal segment of the left anterior descending artery, a myocardial bridge in the middle segment, and 95% stenosis in the middle segment of the left circumflex artery (LCX). A Medtronic Endeavor Resolute stent (2.75 × 24 mm) was implanted, followed by dilatation with a 3.0-mm NC balloon (Figure 1). After the procedure, she was continuously treated with aspirin, ticagrelor, statins, ezetimibe, and bisoprolol; low-density lipoprotein cholesterol was controlled at 0.8–1.5 mmol/L.

**Figure 2:**
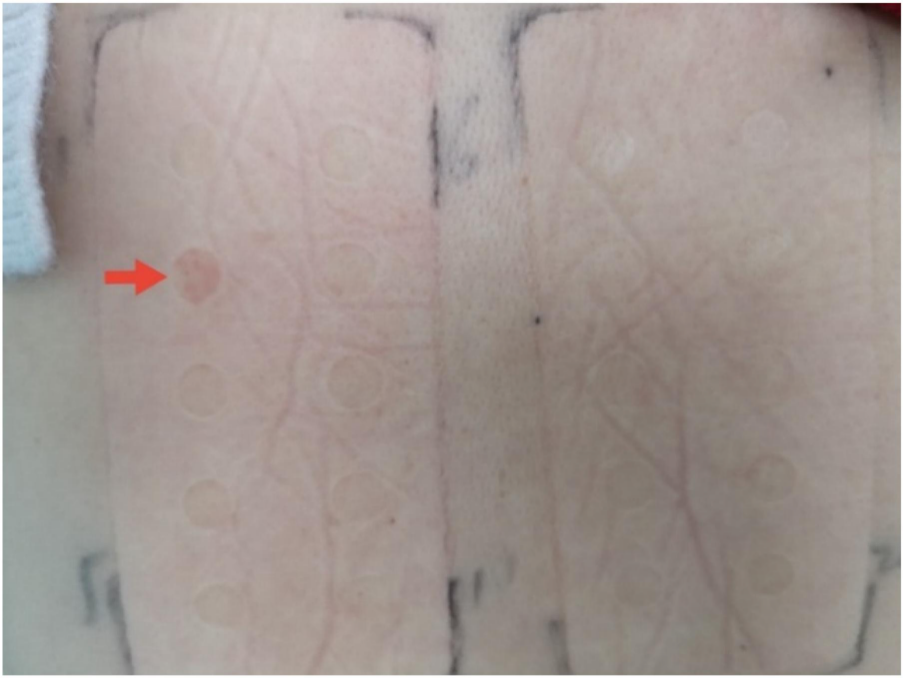
Skin patch test: nickel sulfate allergy was strongly positive 2 + (red arrow).

In March 2021, the patient underwent CAG for acute non-ST-segment elevation myocardial infarction, which showed 50% stenosis of the proximal LCX 95% restenosis in the original stent, and complete occlusion of the first marginal branch opening. Upon enquiring, the patient reported a history of allergy to metal jewelry. When she wore alloy earrings, she developed an abscess reaction in the ear hole, but no obvious allergy when she wore pure gold earrings. The patient was thus suspected of having stent metal allergy. A skin patch test confirmed nickel sulfate allergy (2+, Figure 2), but was negative for cobalt allergy. Nickel allergy was proposed as the reason for stent restenosis. The patient was successfully treated with a drug-coated balloon (DCB) (2.5 × 20 mm).

**Figure 3:**
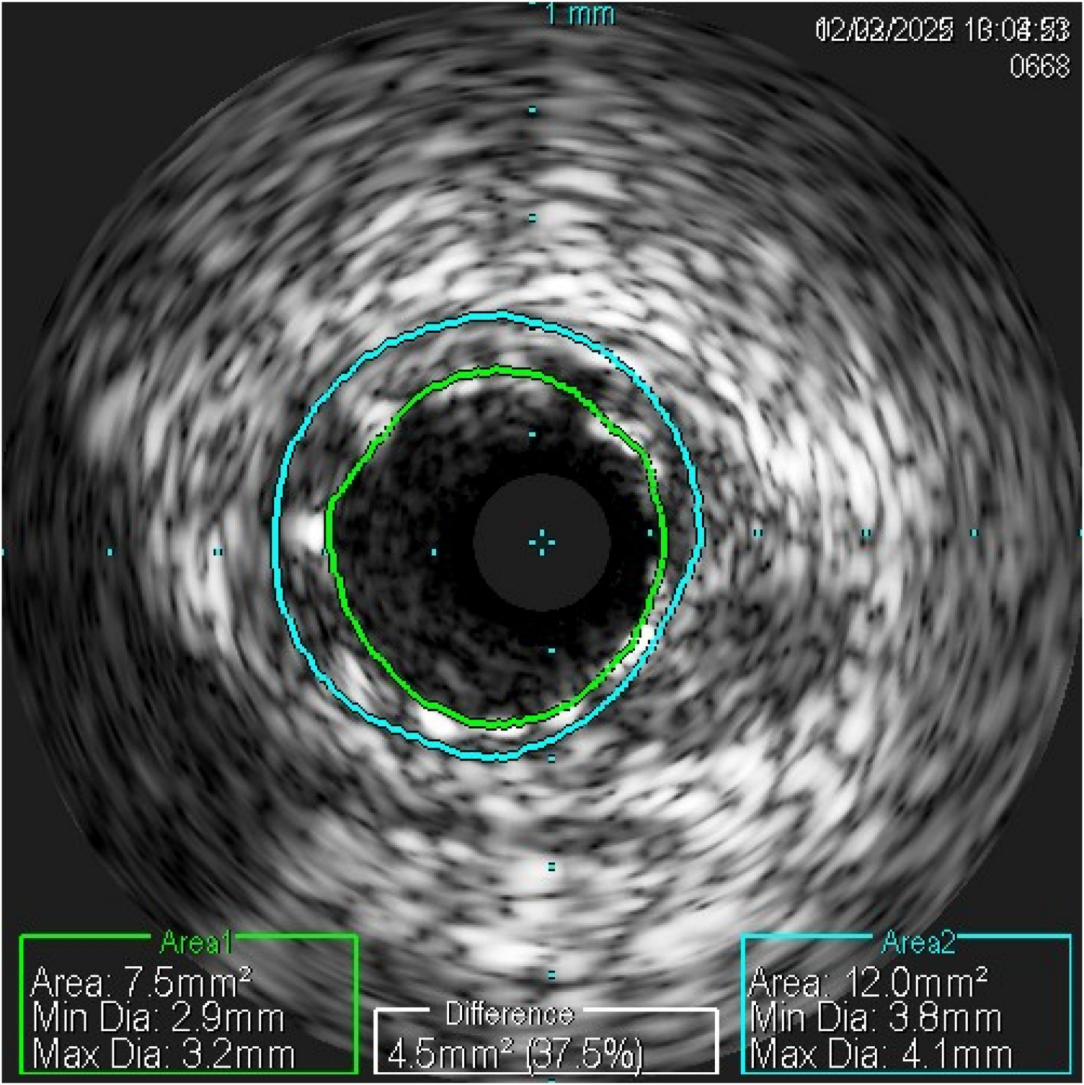
IVUS showed neoatherosclerosis. Minimal stent area was 7.5 mm^2^, minimal stent area/mean reference lumen area was 0.78.

**Figure 4:**
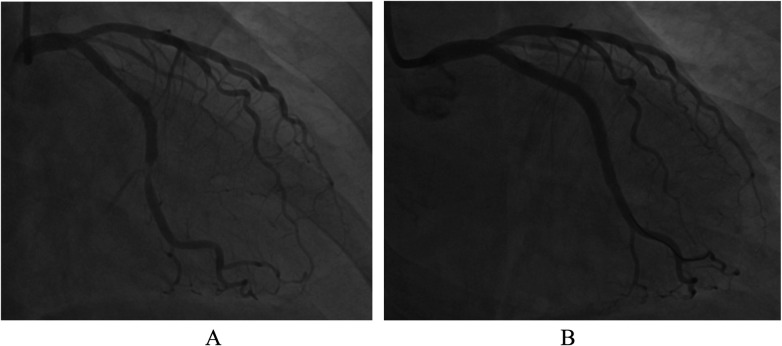
**(A)** CAG before PCI. **(B)** CAG after stenting.

In February 2022, the patient was hospitalized for the third time due to ACS. CAG revealed 50% stenosis in the proximal segment of the LCX, and 95% restenosis at the end of the original stent in the middle segment of the LCX. After predilatation with a 2.0-mm balloon, intravascular ultrasound (IVUS) of the LCX demonstrated neoatherosclerosis in the stent, the original stent was well attached to the wall, the proximal segment had a 76.6% plaque burden rate, and the minimum lumen area was 3.6 mm^2^ on the LCX (Figure 3). This time, a Boston Scientific Promus Premier stent (2.75 × 38 mm) was implanted into the LCX (Figure 4), followed by postdilatation with a 3.0-mm balloon. Repeated IVUS examination confirmed that the stent had adhered to the wall and fully expanded. This platinum–chromium alloy stent was chosen due to its lower nickel content (containing 33% platinum, 18% chromium, and 9% nickel).

**Figure 5:**
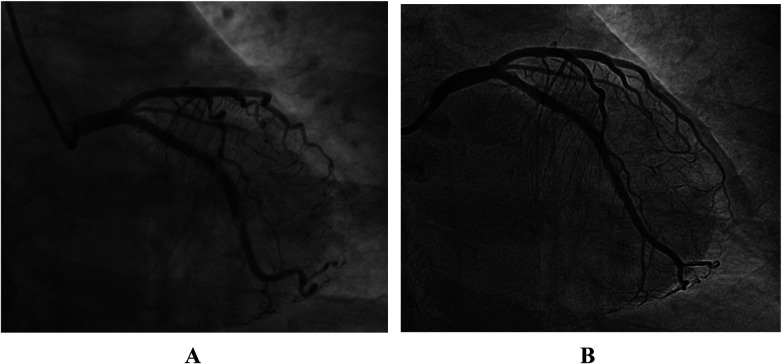
**(A)** CAG before PCI. **(B)** CAG after PCI.

In August 2023, the patient was rehospitalized due to ACS. CAG revealed restenosis on the newly implanted Boston Scientific Promus Premier stent. The patient was successfully treated with DCB (2.5 × 20 mm, 2.75 × 20 mm) (Figure 5). Following discharge, the patient was treated with additional Shexiang Baoxin Pills to treat angina pectoris in addition to her existing medications. The patient has remained angina-free to date. This case report was approved by the ethics committee of Zhongda Hospital, Southeast University.

## Discussion

ISR remains the main limitation of percutaneous coronary stenting, particularly in patients with a history of metal allergy. This case illustrates refractory in-stent restenosis persisting despite various treatment methods, including stents with different metal components, drug-coated balloons, and intensive drug therapy.

This patient was initially implanted with the Medtronic Endeavor Resolute stent, composed of MP35N nickel–cobalt alloy (containing 35% nickel, 35% cobalt, and 20% chromium). After implantation, the first ISR occurred. During detailed inquiry, the patient reported a history of metal jewelry allergy. A skin patch test confirmed nickel allergy, which was considered to be the cause of the patient's ISR. The patient was successfully treated with DCB, but ISR recurred. Eleven months later, a Boston Scientific Promus Premier stent was implanted into the LCX lesion. This stent was made of platinum–chromium alloy (containing 33% platinum, 18% chromium, and 9% nickel) and the nickel content was significantly reduced. However, the patient developed recurrent ISR 18 months later. The patient underwent DCB and was treated with additional Shexiang Baoxin Pills to treat the angina (Table 1) (8). The patient remains asymptomatic till date, with LDL in the target range with lipid lowering medications, no motion abnormality, and normal ejection fraction during repeated echocardiography examinations.

**Table 1:** Timeline of PCI.

Time	PCI	Result
12/2019	Endeavor Resolute stent (Medtronic)	Success
3/2021	DCB	Success
2/2022	IVUS, Promus Premier stent (Boston Scientific)	Success
8/2023	Cutting balloon, DCB	Success

The prevalence of nickel hypersensitivity in the general population is estimated at 8.6%, with higher prevalence in females, typically relating to repeated exposure to consumer items such as jewelry, zip fasteners, and cell phones (9, 10). Although many biological and mechanical factors interplay to produce ISR (11), severe metal stent allergy is seldom a cause of refractory ISR, which lacks effective treatment. Previous studies have reported that nickel allergy may play a role in the pathophysiological process of ISR (12), particularly recurrent ISR (13). However, some studies have demonstrated that metal allergy does not seem to increase the overall risk of ISR (14, 15). Several factors needed attention based on the clinical experience of our patient. First of all, before implanting a coronary stent, patients should be asked about a history of metal allergy and informed of the possible increased risks (7). If patients with a history of metal allergy need to undergo coronary stent implantation, a skin patch test should be performed before the operation to help identify the specific metal they are allergic to, so as to avoid implanting stents containing those metal components. Intravascular imaging guided percutaneous coronary intervention can reduce the risk of ISR. However, the current mainstream stents contain nickel at different content ratios. If the patient’s metal allergy history had been known during the time of her first admission, we might have chosen the Boston Scientific Promus Premier stent as the first choice. However, decision-making in stenting for patients with metal allergy remains unclear. In clinical practice, for patients who develop allergies after stent implantation, the use of antiallergic drugs might delay the progression of ISR (16). Most stents implanted in earlier stent allergy studies were bare-metal stents. The antiproliferative effect of drug-eluting stents (DESs) may reduce the risk of restenosis caused by metal allergies. For patients implanted with DES, a history of metal allergy has not been significantly associated with ISR (4). DCBs provide a stent-free alternative, reducing risks like stent thrombosis and ISR, and are increasingly considered reasonable suggestions for clinical use (17, 18). Indeed, in this case, the patient received DCB treatment in the year 2021, but ISR still occurred following DCB. The patient was since prescribed Shexiang Baoxin Pills to control angina symptoms. Shexiang Baoxin is a traditional Chinese medicine, which previous studies have found to be safe and effective in significantly reducing angina frequency in patients with stable coronary artery disease (19).

IVUS examination ruled out the procedural factors of ISR, such as stent under-expansion, stent malposition, and stent edge dissection in this case. In our case, recurrent ISR developed even after implantation of the low-nickel Boston Scientific Promus Premier stent and subsequent DCB. Therefore, metal stent allergy can be considered a challenging issue in clinical practice. Few studies have reported potential interventions to slow stent restenosis in patients with a history of metal allergy. Intravascular brachytherapy may be useful for refractory ISR (20). Further improvement of stent metal materials and the use of DCB (21) and bioresorbable stents might serve as potential alternative treatment options for patients with metal stent allergy. Clinicians should remain vigilant when evaluating patients with ISR. The major limitations of this report include the diagnostic uncertainty of patch testing, the absence of histologic confirmation, and short follow-up duration. These issues should be considered when interpreting the results of this report.

## Data Availability

The original contributions presented in the study are included in the article, further inquiries can be directed to the corresponding author.
